# Soil fertility interactions with *Sinorhizobium*-legume symbiosis in a simulated Martian regolith; effects on nitrogen content and plant health

**DOI:** 10.1371/journal.pone.0257053

**Published:** 2021-09-29

**Authors:** Franklin Harris, John Dobbs, David Atkins, James A. Ippolito, Jane E. Stewart

**Affiliations:** 1 Department of Agricultural Biology, Colorado State University, Fort Collins, Colorado, United States of America; 2 Department of Forest and Rangeland Stewardship, Colorado State University, Fort Collins, Colorado, United States of America; 3 Department of Soil and Crop Sciences, Colorado State University, Fort Collins, Colorado, United States of America; Government College University Faisalabad, PAKISTAN

## Abstract

Due to increasing population growth and declining arable land on Earth, astroagriculture will be vital to terraform Martian regolith for settlement. Nodulating plants and their N-fixing symbionts may play a role in increasing Martian soil fertility. On Earth, clover (*Melilotus officinalis*) forms a symbiotic relationship with the N-fixing bacteria *Sinorhizobium meliloti*; clover has been previously grown in simulated regolith yet without bacterial inoculation. In this study, we inoculated clover with *S*. *meliloti* grown in potting soil and regolith to test the hypothesis that plants grown in regolith can form the same symbiotic associations as in soils and to determine if greater plant biomass occurs in the presence of *S*. *meliloti* regardless of growth media. We also examined soil NH_4_ concentrations to evaluate soil augmentation properties of nodulating plants and symbionts. Greater biomass occurred in inoculated compared to uninoculated groups; the inoculated average biomass in potting mix and regolith (2.23 and 0.29 g, respectively) was greater than the uninoculated group (0.11 and 0.01 g, respectively). However, no significant differences existed in NH_4_ composition between potting mix and regolith simulant. Linear regression analysis results showed that: i) symbiotic plant-bacteria relationships differed between regolith and potting mix, with plant biomass positively correlated to regolith-bacteria interactions; and, ii) NH_4_ production was limited to plant uptake yet the relationships in regolith and potting mix were similar. It is promising that plant-legume symbiosis is a possibility for Martian soil colonization.

## 1. Introduction

Given the circumstances of climate change, biological contagion, or other events that have potential to wipe out humanity, it is unlikely that humans will be able to remain a single planet species. With human populations growing and space for development and arable land becoming increasingly limited on Earth, off-world agriculture will likely be needed on celestial bodies such as Mars [1]. However, the harsh Martian environment challenges many of the basic tenets of biology found here on Earth. Plants will face some phytotoxicity in regolith (Martian soils), the atmosphere is significantly thinner with a different stoichiometry, temperatures can dip to below -100°C, the lack of atmosphere allows dangerous radiation to affect the planet surface, and plants will be reliant upon limited resources for survival [2]. To support a long-term colony and food production on Mars, it is imperative to establish an on-planet food source capable of feeding its inhabitants [2]. However, Martian regolith presents challenges for plant established and growth, which is especially true for nitrogen (N) availability.

With a lack of critical nutrients in Martian regolith, particularly plant-available N, it will be necessary to find methods to supplement regolith in a cost-effective manner. Regolith is the only available on-site medium for growing plants on Mars. Given that it is not feasible to ship earth soils through space because of weight and cost, soil augmentation appears to be the most viable path forward. Regolith has been analyzed from several rover missions, and surveys have found no traces of plant-available N in regolith. In addition, no known significant organic material on the Martian surface has been identified that could supply plant-available N via microbial mineralization [3,4]. Another problem that requires further testing is the low diatomic N (N_2_) content in the Martian atmosphere. With only around 1.9% of the Martian atmosphere being N_2_, the ability of the N-fixing microbial-plant association to utilize N_2_ gas maybe hindered [5]. On Earth, approximately 78% of the atmosphere is N_2_, making N readily available for plant-microbial symbiotic associations.

Nitrogen in Earth’s soils is partly made accessible by decomposers that mineralize organic N forms to release NH_4_, yet this is not the case on Mars as organic matter and microorganisms responsible for mineralization are lacking [4,6]. Furthermore, on Earth *Rhizobium* spp. form symbiotic relationships with leguminous plant roots to produce NH_4_ from N_2_ gas. The mechanism of rhizodeposition of N through root exudation has been shown in previous studies to provide 3–4.5% of fixed nitrogen from the plant-rhizobium symbiosis to the soil [7]. While *Rhizobium* sp. provide NH_4_ readily available for plants to use in various functions (e.g., amino acid and protein production, DNA, RNA, ATP, chlorophyll) [8]; symbiotic microorganisms are likely lacking in regolith. Though plant-available N is lacking on the Martian surface, plants have been shown to grow in regolith simulant [9]. Prior experiments tested *Lupinus* sp., *Vicia* sp., and *Melilotus* sp. because these are common nodulating species that perform well in traditionally harsh soils. Although Wamelink et al. [9] did not inoculate these plants with their respective *Rhizobium* sp., it was posited as one method to increase plant biomass and regolith N availability over uninoculated regolith. In support of this contention, earlier studies using the JSC 1 regolith simulant have shown that at least one *Rhizobium* spp. can survive in a regolith simulant [10]. Regardless, the symbiotic relationship between leguminous plants and *Rhizobium* spp. is likely needed in materials, such as regolith, in order for both species to successfully thrive.

It is well established that N-fixing bacteria (e.g., *Rhizobium* spp.) allow plants to indirectly acquire atmospheric N for their use and directly deposit excess N in the soil [11]. Host specificity has been observed in some species of N-fixing bacteria, and to ensure symbiosis, plants must be inoculated with their respective N-fixing symbiotes [12]. It is currently unknown if plants will benefit from *Rhizobium* inoculations in the harsh chemical and physical stress conditions of regolith, or if enough N will be synthesized to change regolith N content. In addition, it is not known how different *Rhizobium* spp. will respond in regolith.

If N-fixing bacteria can be used to incorporate atmospheric N to Martian regolith, this could be used as a first step in creating a Martian astroagricultural system. Thus, the objectives of this study were to examine the: 1) relationship of nitrogen fixation and plant-microbe symbiosis of *M*. *officinalis* and *S*. *meliloti* in regolith versus potting soil; and 2) effects this relationship has on plant growth and soil N availability. We hypothesized that an increase in plant biomass would be observed in regolith inoculated with their respective N-fixing bacteria, and excess plant-available N would be deposited in the surrounding regolith via rhizodeposition of exudates, similar to the Rhizobium-legume relationship found on Earth.

## 2. Materials and methods

### 2.1. Bacteria and plant acquisition

Sweet clover (*Melilotus officinalis)* seeds were acquired from Hancock Farm and Seed (https://hancockseed.com/). Sweet clover’s associated N-fixing bacteria, *Sinorhizobium meliloti* strain 1021 was provided by the Ane lab at the University of Wisconsin [13] and stored on yeast extract mannitol slants [1 g of yeast extract, 10 g of mannitol, 0.5 g of dipotassium phosphate, 0.2 g of magnesium sulphate, 1 g of calcium carbonate, and 0.1 g of sodium chloride in 1000 ml of distilled water (DI)] [14].

### 2.2. Bacteria isolation and growth

Single colonies of *S*. *meliloti* cultures were grown on YEM agar plates at room temperature for 7 days. Five replicates were made from a single isolate of the original plate to ensure sufficient inoculum.

### 2.3. Regolith acquisition and experiment design

Regolith was acquired from the Martian Garden (a company that manufactures regolith based off the JSC-1 NASA regolith and the data from Mars rover missions [3,4]; Austin, TX, www.themartiangarden.com). Experiments were conducted in the MMS 1 superfine grade regolith which matched average Mars regolith by 95% in consistency and chemical composition. Potting mix (PRO-MIX^®^, Quakertown, PA) was used as a soil control. The potting mix composition was 75–85% Canadian sphagnum peat moss, with the remaining 15–25% being perlite, vermiculite, dolomitic/calcitic limestone, and a wetting agent. Both regolith and potting soil were sent to the Colorado State University Soil, Plant, and Water Testing Laboratory for nutrient analyses (Table 1). Plastic pots (6.4 cm^2^ x 7 cm tall; T.O. Plastics, Inc., Clearwater, MN) were lined with cheesecloth and filled with 250 g of regolith or potting mix.

**Table 1:** Initial potting mix and regolith properties, and NH_4_ concentrations pre- and post-study.

Property	Potting mix	Regolith
pH	5.8	8.7
Organic material (%)	45.1	0.10
Nitrate (mg kg^-1^)	830	13
Ammonium (mg kg^-1^)	10.7	5.5
Phosphorus (mg kg^-1^)	180	2.4
Potassium (mg kg^-1^)	1110	218
Iron (mg kg^-1^)	130	3.8
Copper (mg kg^-1^)	7.1	0.3
Zinc (mg kg^-1^)	8.4	0.2
Boron (mg kg^-1^)	0.5	0.8
Manganese (mg kg^-1^)	48	0.6
Bulk density (g cm^-3^)	0.16	1.35
**Treatment**	**Pre-study NH**_**4**_	**Post-study NH**_**4**_
	------------------------------ (mg kg^-1^) ------------------------------	
Potting mix inoculated	10.7	11.2±5.2
Potting mix uninoculated	10.7	3.31±0.8
Regolith simulant inoculated	5.50	3.7±1.2
Regolith simulant uninoculated	5.50	2.6±0.9

### 2.4. Plant germination and experimental design

Seeds were treated in 250 mg L^-1^ gibberellic acid in petri dishes for 5 minutes to aid in uniform germination prior to sowing, with two seeds sown into each pot. After 2 weeks, each pot was thinned to one plant per pot. If both seeds failed to germinate in a pot, seedlings were transplanted from pots with duplicates with care taken to limit root damage and observed for transplant shock before inclusion. Ten pots of both potting mix and Mars regolith were inoculated with *S*. *meliloti* (see section 3.5 below), while five pots of both potting mix and Mars regolith remained uninoculated as controls. Each pot was spaced 10 to 15 cm apart, with placement of each pot completely randomized across the bench. Plants had consistent light from grow lights in the CSU greenhouse and the ambient temperature was kept between 25 and 30°C. Plants were watered with 80 ml sterile DI water every other day except for days they were inoculated.

### 2.5. Plant inoculation and maintenance

Inoculum was prepared by adding 10 ml of sterile DI water to YEM agar plates containing *S*. *meliloti* cultures, and hand mixing bacterial cells into a solution with a sterile glass scraper. The aqueous inoculum was added to 500 ml of sterile DI water and mixed with a sterilized stirrer for 3 minutes at room temperature. The inoculum was enumerated using a Bio-Rad SmartSpec Plus Spectrophotometer (Bio-Rad Laboratories, Inc, Hercules, CA). Inoculum was diluted to 5x10^8^ cells ml^-1^ [15]. Plants were inoculated after five weeks from sowing with 80 ml of inoculum; controls (i.e., no inoculum) were watered with 80 ml of sterile DI water. Afterwards, all plants were watered with 80 ml of sterile DI water 3 times per week until harvest. Water was allowed to drain through the pot while cheesecloth was used to prevent the media inside from being lost through the drainage holes.

### 2.6. Plant harvest and evaluation

Plants were harvested three months following inoculation. Whole plants were carefully removed from their growth medium and measured above and below the root sheath for shoot and root lengths, respectively. Nodules were numerated for each plant and were visually observed for a reddish color that has been associated with nodule health [16]. Plants were then cut at the root sheath and shoots and roots were dried at 60°C for 48 hours, and then all plant materials were weighed.

### 2.7. Soil nitrogen and soil fertility testing

Following plant harvest, soil from each pot was collected and frozen at -80°C until further use. At the time of analysis, soils were thawed and NH_4_ was extracted using a 2M KCl solution (5g soil:50 ml extracting solution) and analyzed colorimetrically using the salicylate-nitroprusside method [17]. NH_4_ in each soil sample was compared to the NH_4_ concentration from the background regolith and potting soil to determine how much NH_4_ was absorbed by plants or added to the media via *Rhizobium*.

### 2.8. Statistical analysis

Statistical analysis was performed in R [18]. One-way ANOVA or Student’s T-tests were used for within group testing to examine differences between treated and control samples for each potting mix and regolith. Two-way ANOVA tests were used to compare variance and differences between potting mix uninoculated/inoculated groups and regolith uninoculated/inoculated groups. In addition, two-way ANOVA tests were used to compare growth media NH_4_ concentrations and shoot and root biomass ratios across treatments. Linear regression analyses were used to determine if relationships between nodule quantity, plant biomass, and NH_4_ occurred in the treatments.

## 3. Results

### 3.1. Prestudy soil status

Fertility analyses on potting mix and regolith prior to addition of plants or microbes was conducted by the soil testing lab at Colorado State University (Table 1). Potting mix had a greater nutrient content when compared to regolith, especially with respect to NO_3_ and NH_4_ concentrations. Nutrients were, on average, 32.6 ± 9.8 times more abundant in potting mix than in regolith.

### 3.2. Plant growth

**Fig 1:**
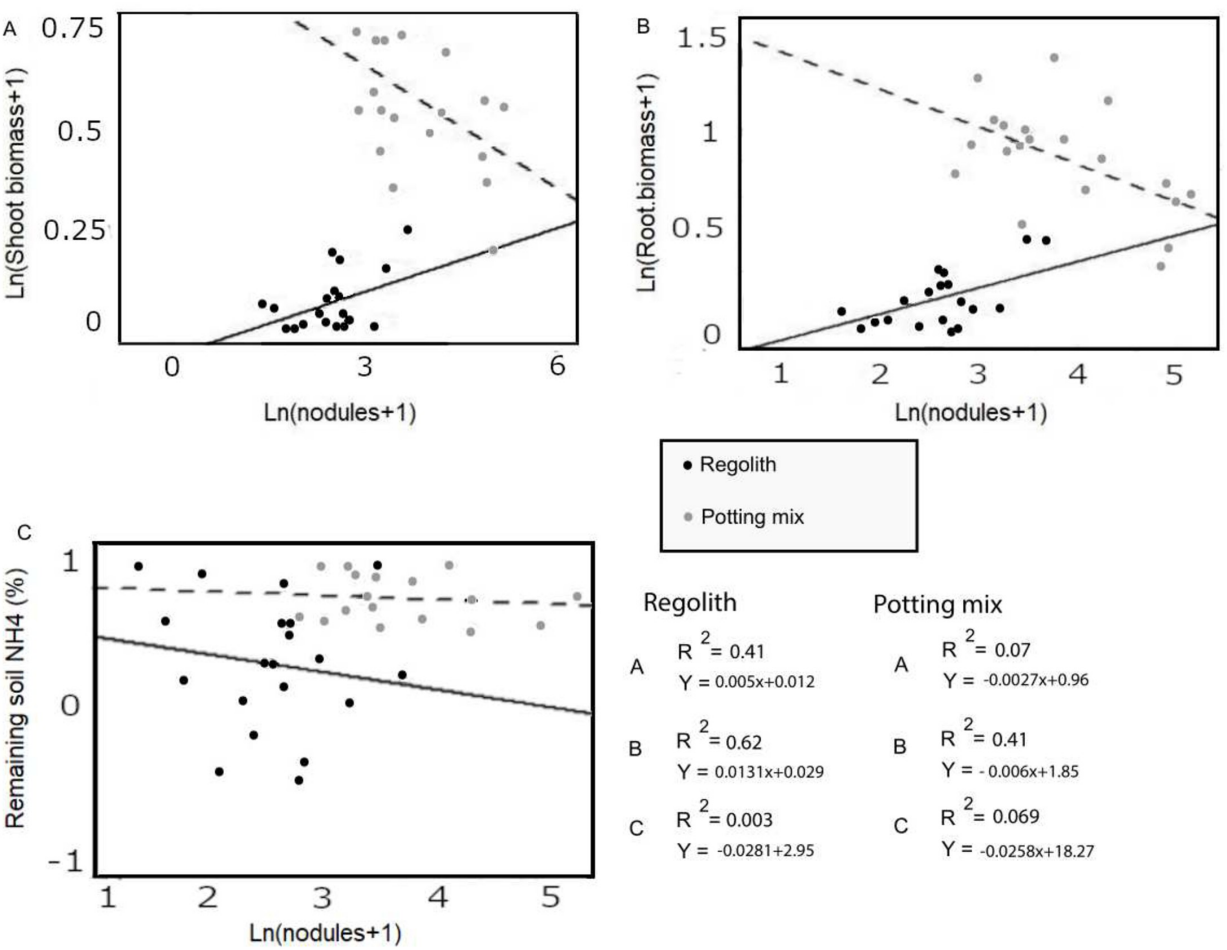
Linear regression of shoot biomass by nodules (A), root biomass by nodules (B) and remaining and added nitrogen in soil by quantity of nodules (C) for either regolith or potting soil. NH_4_ was calculated using the pre-experiment sample for each group and creating a percent for each data point. Data were checked for normality; outliers were removed, and the log scale of the data was taken.

Significant differences in shoot length, shoot biomass, and root biomass were observed within regolith and potting mix uninoculated and inoculated groups (Fig 1). Plant shoot lengths were 2.5 times longer in inoculated plants in each media type compared to uninoculated plants. Plant shoot and root biomass more than doubled in inoculated versus uninoculated treatments in both potting mix and regolith. Overall plant biomass in the potting mix was also significantly greater than in the regolith between both inoculated and uninoculated groups.

### 3.3. Nodulation

Nodules were significantly more abundant on plants in potting mix compared to those in regolith (*P* = 0.00018). Inoculated plants in regolith had on average 14.5 ± 1.9 nodules compared to the inoculated plants in potting mix that had on average 63 ± 10.6. As a control check, uninoculated plants had no nodule formation.

### 3.4. Pre and post study soil NH_4_ concentrations

Neither potting mix nor regolith showed a significant difference in NH_4_ concentration between inoculated and uninoculated groups (regolith: *P* = 0.29; potting mix: *P* = 0.21; Table 1). The average post-study NH_4_ concentration was greater in the inoculated potting mix as compared to the pre-study NH_4_ concentration. However, the average NH_4_ concentration for uninoculated potting mix, inoculated regolith and uninoculated regolith was lower in the post- as compared to the pre-study.

### 3.5. Linear regression analysis of plant growth parameters and nodulation

**Fig 2:**
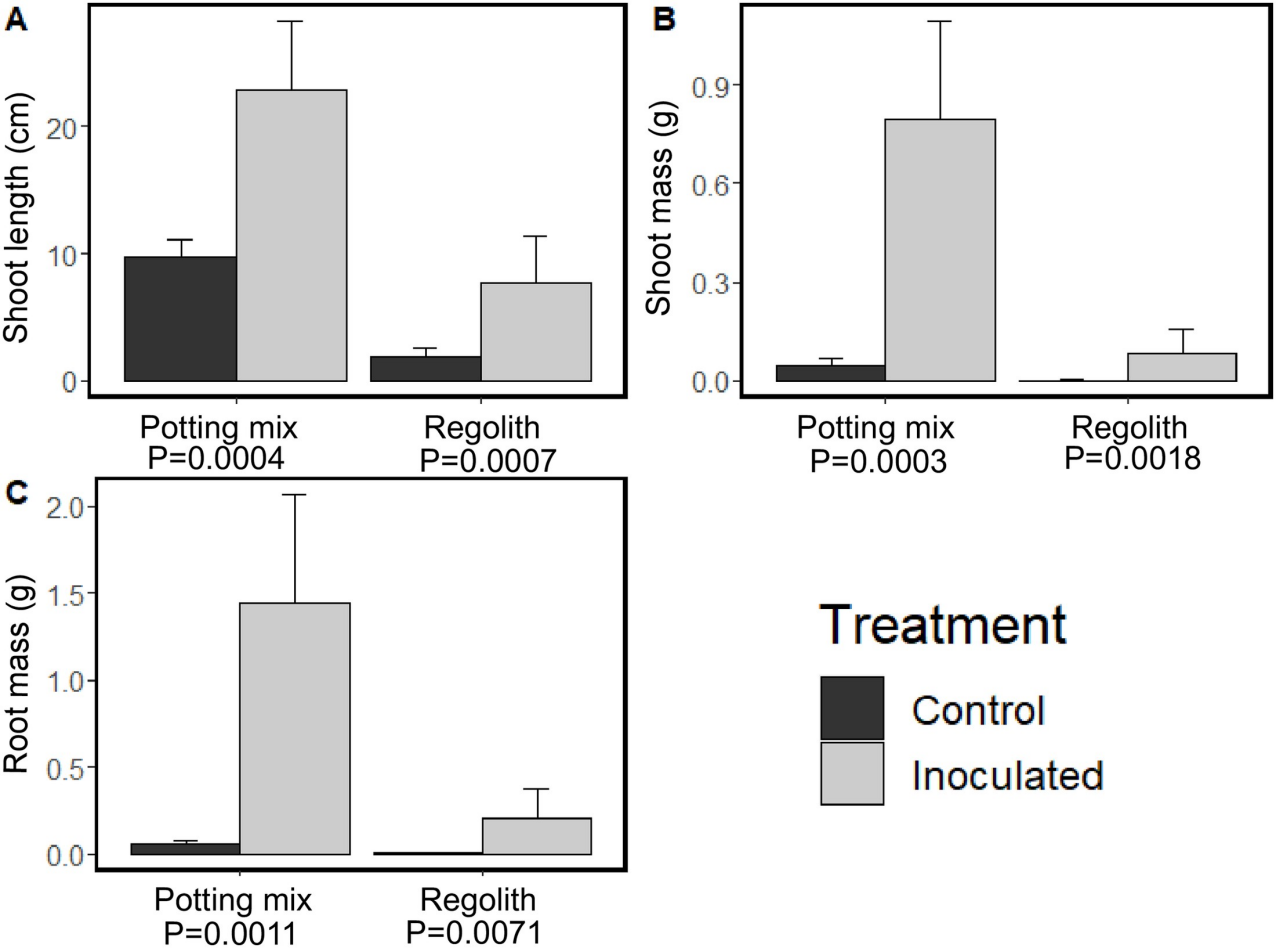
Sweet clover (*Melilotus officinalis)* shoot length (A), shoot biomass (B), and root biomass (C) as measured either above or below the root sheath. Error bars represent standard error of the mean. P-values derived from one-way ANOVA analyses.

Linear regression of shoot biomass, root biomass, and the remaining and added N present in the soil as a function of the number of root nodules is presented in Fig 2. In both media treatments, both root and shoot biomass for inoculated and uninoculated groups fit a linear regression when using root nodules as the predicting factor. The R^2^ values were 0.41 and 0.62 for regolith shoot and root biomass, respectively, while the R^2^ values were 0.07 and 0.41 for the potting soil shoot and root biomass, respectively. These R^2^ values indicate positive and negative relationships between plants and symbionts in the regolith and potting mix, respectively. The R^2^ values of remaining NH_4_ plotted as a function of nodules showed very little correlation with respect to R^2^ values for potting mix (0.0691) and regolith (0.0033; Fig 2).

## 4. Discussion

This research highlights the importance of using naturally forming partnerships between plants and their symbiotic bacteria to increase plant growth success in regolith, one of the first steps towards understanding the capability of establishing astroagricultural colonies on Mars. Though clover (*Melilotus officinalis*) had been previously demonstrated to grow in regolith [9], our study found that plant shoot and root growth was increased by over 75% when inoculated with *S*. *meliloti* compared to plants grown in uninoculated regolith. Our study highlights the importance of nitrogen as a major limiting factor for plant growth in regolith, suggesting that nitrogen-fixing bacteria can be used to reduce this limitation. Though we have demonstrated this using clover, this research may be the foundation for future research on other food producing crops.

Rhizobia significantly enhanced plant growth in the regolith, suggesting that nitrogen is the major limiting resource for plants in this media. It has been shown that *Rhizobium*’s survival and potential to fix nitrogen can be limited by soil stress [19,20]. In this study, effects of soil stress were demonstrated as the potting mix contained significantly greater NH_4_ concentrations after plant growth, though plants in potting mix were not initially limited by nitrogen. At the end of the experiment, the mean difference in NH_4_ between regolith and potting mix inoculated groups was 7.5 mg kg^-1^ (Table 1).

The limitation of the symbiosis to produce reactive nitrogen demonstrates previously studied challenges to the viability of Martian regolith as a in situ resource for agriculture. Chemical stress from Martian regolith has been shown to be extremely detrimental to plants [21]. The same study showed that without the addition of nutrients, *Arapodobsis thaliana* died within 10 days of germination [21]. However, when Hoaglands No. 2 nutrient solution was added, plants only experienced about a 10% die off. Additionally, while most simulants of Martian regolith are able to support plant life with the addition of nutrients and acidification, no current simulants account for the calcium perchlorate deposits on the Martian surface [21]. While this study is a step to correcting for nutrient deficiency and move in the direction of terraforming Martian regolith, more studies including phytoremediation or mycoremediation will be needed to correct for other toxicity issues in Martian regolith.

While the above challenges remain, when in regolith, the addition of captured atmospheric nitrogen likely increased N for the plant, therefore decreasing this as limiting factor on plant growth [22]. However, despite less nitrogen being fixed in the regolith, NH_4_ appeared more important for plant growth than in the potting mix because of its increased scarcity. The presence of nitrogen is required for nodulation and establishment of rhizobium, yet it is commonly used up by plants in low N environments, leading to N deficiency in the plant [23]. Micronutrient content could be another restrictive property as the regolith lacked Fe, Cu, Zn, and Mn. Although not analyzed in the regolith, Mo may have also been lacking as it is important for nitrogen fixation [24]. In essence, plants in the potting mix were likely not limited by soil nutrient deficiencies as compared to the regolith.

We expected that in N-poor regolith, more root nodules would have been formed as compared to the potting soil, yet the opposite was observed. A relative reduction in regolith nodulation may have caused by other limitations such as pH or deficiency in almost every plant nutrient (Table 1), and in particular, available Fe. Prior research established that plants with nodules require more Fe to sustain their relationship with rhizobium [25]. In addition, the rate of N fixation by rhizobia in some plants is positively correlated to available soil Fe concentrations [26]. Furthermore, the simulated regolith pH was fairly high (8.7), and thus may have impaired plant Fe intake, further reducing nodule formation and nitrogen fixation (Table 1). Martian soils have between 5 and 14% iron oxide [27] but a soil pH of ~ 8.0 [28]. Given these conditions, plant-available Fe content would likely be less than 10^−24^ M Fe^3+^ and thus low, if not lower, than the initial regolith Fe concentrations [29]. Overcoming challenges in plant nutrient availability will need to be considered in order to effectively grow plants on Mars.

With respect to plant biomass, the number of nodules had a negative relationship with potting mix plants and a positive effect with regolith plants (Fig 2). Negative correlations between nodule formation and potting mix may have resulted from the presence of pre-existing nitrate. In nodulation, plants generally form associations with rhizobium at lower rates when nitrate (and/or NH_4_) is abundant [30]; yet, plants grown in potting mix had significantly more nodules than regolith. Two likely explanations of this could be that nodules formation resulted in less biomass, or that available N at the initial condition in potting mix could have increased plant health and growth at the beginning of the experiment rendering nodules less effective. Interestingly, there was also less remaining NH_4_ per nodule in the potting mix (Fig 2). This may be explained by the larger size of the plants in the potting mix, as they were not limited by the other restrictive properties (compared to the regolith) and were able to use more available nitrogen. Potting mix nitrate could have leached over time due to watering, and it is also possible that plants may have assimilated nitrate prior to leaching and other normal soil N cycling processes [31–33]. Without measures of nitrate at the conclusion of the study, it is not possible to discern the cause, however it was not crucial to the main hypothesis this study tested.

Soils commonly lose nitrate to leaching [34]. Nitrate is transformed from NH_4_ when NH_4_ is converted by nitrifying bacteria [35]. While the primary form of nitrogen that results from plant-bacterial symbiosis is NH_4_, in terrestrial soils nitrifying bacteria convert NH_4_ to nitrite or nitrate. Because Mars has no bacteria observed in its regolith, there would likely be no *Nitrosomonas* or *Nitrobacter* to convert NH_4_ to nitrite and nitrate, respectively. The loss pathway for nitrite and nitrate is most commonly leaching, while the leaching loss of NH_4_ would likely be less of a concern when watering [36]. This could prove beneficial when raising and irrigating plants in regolith. However, given the high regolith pH (7.8), ammonia volatilization would likely be a more significant loss pathway concern on Mars. Ammonia volatilization occurs to greater extents as soil pH becomes more alkaline [37], increasing almost linearly above pH 8 [8]. However, over the long-term, ammonia volatilization drives pH down, and in the case of regolith, could make soils more suitable for plants and rhizobia [38].

Study results showed the symbiosis benefited plant growth and phenology, in both regolith and potting mix. Though we know that rhizodeposition occurs, NH_4_ did not appear to accumulate in the soil. N-starved plants likely used all available soil NH_4_. Further, the lack of plant decay likely kept sequestered available N in plant roots. Given that the plants were not used as green fertilizer, the sequestered N was never released back into the soil. Thus, deposition from decay was not possible. An insufficient amount of experimental time could have been another factor as to why soil NH_4_ concentrations did not increase, as suggested by others using regolith [39]. Companion cropping by using nitrogen fixing rhizobium and their plant symbiotes generally occurs at one year intervals [40]. In these cases, root exudation and root die off that result in subsequent release of nitrogen are thought to be integral to the transfer of nitrogen in these systems [42]. In order to overcome this issue in future studies, more plants per pot could be added to increase the amount of N fixation that occurs per volume of regolith and the experiment could be run for a longer duration. An additional option for increasing regolith N and its N storage capacity would be to till nitrogen containing plants, like clover, into regolith as green fertilizer [41,42], along with the addition of decomposer microorganisms to produce more bioavailable nitrogen via mineralization [6,43]. One study reported that the addition of organic matter in regolith, using grass clippings from *Lolium perenne* L., resulted in an improvement in plant growth displayed in plant phenology as plants grown in previous studies did not show seed or fruit production [44]. As fungi and bacteria are routinely placed in cold storage for archiving and research purposes, this process could be replicated for transport to Mars. Plant incorporation and decomposer cryogenesis/revival, followed by regolith application, should be quantified on Earth before use on Mars.

Plants generally cannot grow without accessible nitrogen, and can only grow poorly in areas with scarce nitrogen [23]. However, the study by Wamelink et al. [9] showed that plants, other than nodulating plants, could grow in regolith, though the authors had a difficult time germinating seeds of nodulating plants. In comparison to nodulating plants used in Wamelink et al. [9], this current study showed nodulating plants inoculated with their respective *Rhizobium* sp. were able to survive for longer time periods. For comparison, the average *M*. *officinalis* survival rate after 50 days was roughly 50% as observed by Wamelink et al. [9]; whereas in our study, after 90 days, 100% of inoculated plants survived. Increasing study duration would aid in filling in gaps about persistence of plants in harsh conditions.

While *Rhizobium* spp. fix atmospheric N_2_, regolith also contains bioavailable P and K, as well as some other micronutrients [3,4]. However, other methods of fixing or adding missing micronutrients will be needed for those which are not present. Specifically, Cu, B, and Mo are not present in regolith based on Mars rover analysis [3,4]. Another considerable issue that requires attention is how the atmospheric composition and density of Mars affects plant growth, plant gas exchange and ultimately N fixation. Mars has 31 times less atmospheric N at equal density than Earth. It seems prudent to test whether a condensed atmosphere of that composition would be able to support rhizobia N fixation [5]. Plants would likely have to be grown in a biosphere—an enclosed area with artificial heat and light. While a biosphere would be a necessity, it is unclear if only atmospheric composition would need to be altered or if also atmospheric density, as altering the stoichiometry of an enclosure could be energy taxing. The drastic difference between the stoichiometry of terrestrial and Martian atmosphere N content (78% versus 1.2%, respectively) may be pivotal for *Rhizobia* spp. and their ability to fix atmospheric N. A possible way to overcome this, should it be an issue, would be to breed plants and symbiotes for low N atmospheres.

Interestingly, the lack of *Nitrosomonas* spp. and *Nitrobacter* in regolith would likely keep bioaccessible N in the form of NH_4_, with it not being converted to nitrate. Future experiments could focus on whether nitrification is a benefit to plants or if nitrifying bacteria addition is beneficial for N cycling in regolith. As observed at the end of our study, little NH_4_ was remaining in regolith, and based on plant growth there was likely N within the plant, although this was not determined; future tissue quality analysis with inoculated and uninoculated plants could confirm this concept and could provide invaluable data for astroagricultural success on Mars.

## 5. Conclusion

Martian colonization will be increasingly needed in the future, yet additional soil and atmospheric augmentation research will be required to develop astroagricultural techniques and allow for the greatest probability of success in Martian farming. This research demonstrates that based on regolith properties and its limited nutrients, Rhizobia can significantly increase plant growth in regolith. In addition, the relationship between *Rhizobium* spp. and plants differs when comparing regolith to soils; regolith interactions were positively correlated to plant biomass. Additional research focused on augmenting regolith would serve to reduce remaining ambiguities and to provide a broader understanding of how plants would function within Martian planetary dynamics.

## Supporting information

S1 Table (XLSX)
